# Waste Management in the Agri-Food Industry: The Conversion of Eggshells, Spent Coffee Grounds, and Brown Onion Skins into Carriers for Lipase Immobilization

**DOI:** 10.3390/foods11030409

**Published:** 2022-01-30

**Authors:** Sandra Budžaki, Natalija Velić, Marta Ostojčić, Marija Stjepanović, Blanka Bilić Rajs, Zita Šereš, Nikola Maravić, Jovana Stanojev, Volker Hessel, Ivica Strelec

**Affiliations:** 1 Faculty of Food Technology Osijek, Josip Juraj Strossmayer University of Osijek, Franje Kuhača 20, 31000 Osijek, Croatia; nvelic@ptfos.hr (N.V.); mostojcic@ptfos.hr (M.O.); marija.nujic@ptfos.hr (M.S.); blanka.bilic@ptfos.hr (B.B.R.); ivica.strelec@ptfos.hr (I.S.); 2 Faculty of Technology Novi Sad, University of Novi Sad, Bulevar Cara Lazara 1, 21000 Novi Sad, Serbia; zita.seres@uns.ac.rs (Z.Š.); maravic@uns.ac.rs (N.M.); 3 BioSense Institute, University of Novi Sad, Dr Zorana Djindjica 1, 21000 Novi Sad, Serbia; jovana.stanojev@biosense.rs; 4 School of Chemical Engineering and Advanced Materials, North Terrace Campus, The University of Adelaide, Adelaide 5005, Australia; volker.hessel@adelaide.edu.au

**Keywords:** eggshells, spent coffee grounds, brown onion skins, conversion techniques, enzyme immobilization

## Abstract

One of the major challenges in sustainable waste management in the agri-food industry following the “zero waste” model is the application of the circular economy strategy, including the development of innovative waste utilization techniques. The conversion of agri-food waste into carriers for the immobilization of enzymes is one such technique. Replacing chemical catalysts with immobilized enzymes (i.e., immobilized/heterogeneous biocatalysts) could help reduce the energy efficiency and environmental sustainability problems of existing chemically catalysed processes. On the other hand, the economics of the process strongly depend on the price of the immobilized enzyme. The conversion of agricultural and food wastes into low-cost enzyme carriers could lead to the development of immobilized enzymes with desirable operating characteristics and subsequently lower the price of immobilized enzymes for use in biocatalytic production. In this context, this review provides insight into the possibilities of reusing food industry wastes, namely, eggshells, coffee grounds, and brown onion skins, as carriers for lipase immobilization.

## 1. Introduction

The development of a new generation of immobilized (heterogeneous) biocatalysts is a priority in line with the growing interest in industrial products obtained in a healthy and sustainable way [1], regardless of the following product groups: food, chemicals (biofuels), or pharmaceuticals. Indeed, it is expected that new and/or improved existing technologies will be needed in the coming decades to follow the trend towards sustainable production and to allow better utilization of waste from the agri-food industry, for which all fossil reserves are still exploited today. Selected recent studies presented in this review confirm that there is no lack of potential for the above-mentioned topic. Replacing chemical catalysts with biocatalysts, and particularly immobilized (heterogeneous) ones, can address outstanding issues of energy efficiency, environmental friendliness, and cost-effectiveness of the process.

The study on the life cycle assessment of biodiesel production in relation to the use of different catalysts shows that the negative environmental impact of biodiesel production decreases when heterogeneous biocatalysts (immobilized lipases) are used compared to the use of chemical catalysts or homogeneous biocatalysts, mainly due to their repeated use. At this stage, the degree of environmental impact strongly depends on the number of reuses of heterogeneous biocatalysts during the production process [2]. This is true not only for biocatalytic biodiesel production, but also for any other biocatalytic production that seeks to implement the principles of sustainable development.

Sustainable production, from the introduction of the concept and definition by Agenda 21 to the present [3], represents a development that meets the needs of current generations without compromising the ability of future generations to meet their needs [4]. It should be noted that behind this definition there are not only activities related to environmental protection but also a whole range of activities related to the protection of natural, cultural, and social values that are closely linked to material goods. Sustainable development thus stands for a series of technical, technological, economic, and social changes carried out to meet the needs of the present and future generations. One of the pillars of sustainable development is the circular economic model, which aims to ensure sustainable development at every stage of product creation, processing, and transformation by creating a “closed-loop” economy [5]. The transition from a linear to a circular economy model means that resources remain in the economy even after the end of the life cycle of materials and products. The new value is created through a “closed loop”, thus approaching a model that avoids the creation of waste, i.e., the “zero waste” model [6].

One of the five major challenges of sustainable waste management in the agri-food industry using the “zero-waste” model [7] is the development of innovative waste utilization techniques for the production of chemicals, fine chemicals, bioactive compounds, enzymes, and functional materials. These products have, at least, twice the added value of products derived from the currently prevailing, outdated waste management strategies that are not consistent with sustainable development. Outdated waste management strategies result in low value-added products, such as animal feed, treated waste from the processes of composting, anaerobic digestion, and incineration, which can also have a negative impact on the environment and ultimately, and most undesirably, result in landfilling [8].

Previous research has shown that there is a possibility to use waste oils and fats from the agri-food industry as cheaper feedstock [9,10,11,12] for biodiesel production. In addition, solid waste from pumpkin oil production can be used as a carrier for the immobilization of lipases for sustainable biocatalytic production of biodiesel [13]. In addition, it has been shown that agri-food industry wastes have the potential for the production of very valuable semi-finished products and products for further application in the food, biotechnological, and pharmaceutical industries [14], and that lipases can be used to treat wastes from the oil industry [15]. Through an economic analysis of biocatalytic biodiesel production in microstructured reactor systems, several authors [13,16,17] have shown that the share of biocatalyst (immobilized lipase) production costs is about 38.5% of the total biodiesel production costs in such systems. Up to 87.9% of the total costs associated with lipase immobilization are associated with the commercial immobilization carrier. This percentage can be significantly reduced if cheaper carriers (commercial and/or alternative) such as agri-food wastes and/or by-products are used and if the immobilized lipase is used in multiple production cycles.

Agri-food wastes/by-products are widely available and inexpensive, and their use in the preparation of the heterogeneous biocatalyst would have the following multiple positive effects: reducing the environmental impact and lowering the cost of the enzyme immobilization phase, which would ultimately lower the price of the final product. The idea of developing carriers based on agri-food waste materials for the immobilization of lipases can be traced back several decades when such waste was primarily used for energy and fine chemical production. However, after being used for the production of fine chemicals, there was always a certain amount of transformed waste material left to be disposed of. Since these transformed materials were mostly lignocellulosic, the idea of using them as carriers for the immobilization of enzymes emerged. Two reviews [18,19] have recently presented a systematic review of studies dealing with lignocellulosic and non-lignocellulosic wastes used for enzyme immobilization. They can serve as a good basis for the analysis of other types of such waste that have not yet found wider application in enzyme immobilization. Recent trends in enzyme immobilization are aimed at using materials that have never been used to obtain immobilization carriers or at finding new uses for already known carriers. Of course, such trends require the improvement of existing and the development of new biocatalytic technologies [20].

In this context, this review provides insight into the potential for reuse of less commonly considered agri-food wastes as carriers for lipase immobilization, focusing on eggshells, coffee grounds, and brown onion skins.

## 2. Lipases as Heterogeneous Biocatalysts

Lipases (EC 3.1.1.3) are triacylglycerol acyl hydrolases that act on carboxylic acid ester bonds. In addition to their natural function of hydrolyzing triacylglycerol to diacylglycerol, monoacylglycerol, glycerol, and free fatty acids, lipases can catalyze esterification, interesterification, and transesterification reactions in non-aqueous media. This versatility has enabled the use of lipases in the food, pharmaceutical, leather, textile, cosmetics, and paper industries, where they have been used in “free” soluble form (homogeneous biocatalyst) or immobilized form (heterogeneous biocatalyst) [1,21,22,23,24,25].

Lipases are used in free form as an active ingredient in foods, such as bakery and pasta products, and various types of cheese and dairy products to improve the quality and taste of the final product [24,26,27,28] or in immobilized form to provide a substitute for human milk [27,28], cocoa butter [29,30,31,32], specific flavors as food additives [33], or for the production of ω-3- and ω-6-fatty acids from fish and other oil species [34,35]. Although lipase accounts for about 10% of all industrial enzymes used in current industrial production, the use of these enzymes is expected to increase due to their versatility [24,36,37,38,39]. Increasing use of immobilized lipases is expected, especially in the chemical industry for the fast-growing field of “green” biodiesel synthesis [38], but also in the processing/pretreatment of wastewater from the food industry with a high-fat content [36,37,39].

**Figure 1:**
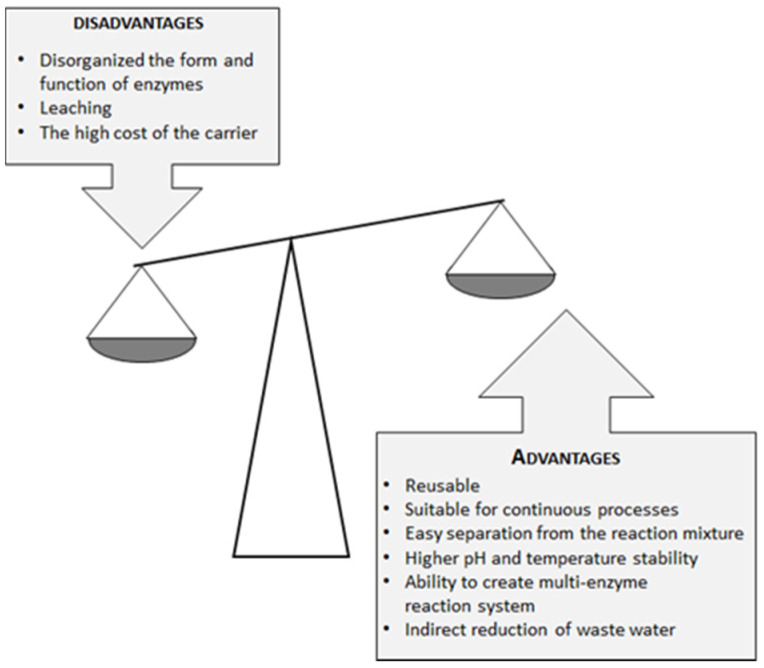
Advantages and disadvantages of heterogeneous biocatalysts (modified according to Ref. [45], with permission from publisher Josip Juraj Strossmayer of Osijek, Faculty of Food Technology Osijek, 2019).

As already stated, the use of biocatalysts (including lipases) compared to chemical catalysts in industrial production is desirable due to high substrate and reaction specificity, environmental compatibility, and lower energy requirements, all of which have an impact on reducing the cost of the production process. The high price of biocatalysts, as well as the very low possibility of reusing the homogeneous biocatalyst, are major drawbacks for wider use of lipases in industrial production. The above problems can be overcome by using immobilized/heterogeneous lipases, which can be easily separated from the reaction mixture after completion of the production process and reused in the continuous production processes. According to literature data, immobilization also improves the operational stability of lipases and the possibility of their use under extreme pH and temperature conditions [40]. Figure 1 shows the advantages and disadvantages of heterogeneous biocatalysts. Immobilized lipases open the possibility of developing new production technologies, e.g., via the transition from batch to continuous processes. By introducing continuous processes instead of batch processes, reactor size and investment costs are reduced, facilitating process control with low variations in product quality. Within the concept of continuous biocatalytic reactors, special attention is paid to microstructured reactors [41,42], as they offer the significant advantage of intensifying mass and heat transfer as so-called “novel process windows” [43,44].

**Figure 2:**
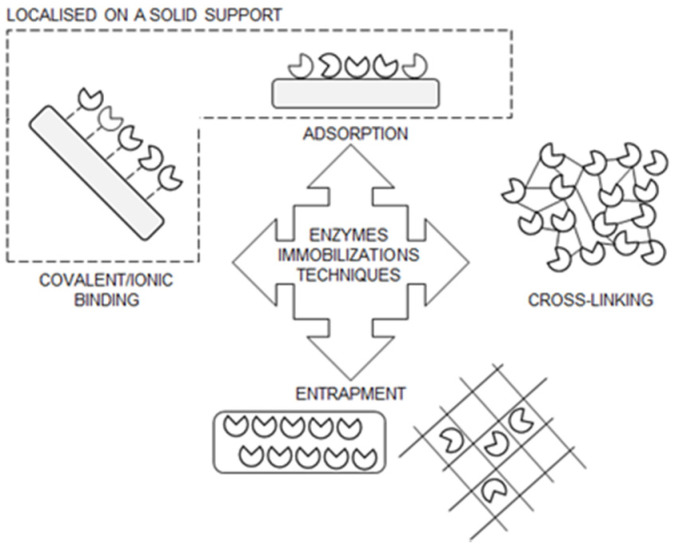
Overview of techniques for the immobilization of enzymes (modified according to Ref. [45], with permission from publisher Josip Juraj Strossmayer of Osijek, Faculty of Food Technology Osijek, 2019).

Immobilization of enzymes means that the enzyme is enclosed in a phase (matrix/support) that is different from the phase of substrates and products. In some cases, Immobilized enzymes entrapped or localized on a solid carrier retain their catalytic activity so that they can be used repeatedly and continuously, while in other cases immobilization improves their catalytic and operational properties. Various immobilization techniques have been used to immobilize enzymes (Figure 2).

Depending on the molecular forces that occur between enzymes and solid carriers, immobilization techniques can be broadly divided into the following: (i) adsorption, (ii) covalent bonding, and iii) entrapment. Adsorption is the simplest method of immobilization based on weak forces (interactions) between the matrix and enzymes, which include van der Waals forces, hydrophobic interactions, and hydrogen bonding, as well as stronger ionic interactions. The latter is more desirable for industrial applications because immobilized enzymes cannot be used indefinitely due to loss of activity upon reuse, but can be stripped (desorbed) from carriers and replaced by “healthy” enzymes under optimal conditions. However, enzyme immobilization by adsorption, which relies on ionic interactions between enzymes and carriers, has yet to find its applicability in industrial production [46].

Covalent binding of enzymes is an effective immobilization technique and has been shown to be the most stable interaction for the immobilization of enzyme molecules to the carrier. Covalent binding usually involves the binding of amino groups of lysine residues to aldehyde, carboxyl, or epoxide groups of carriers, or of carboxyl groups of glutamic acid residues to the amino groups of carriers. The enzymes can bind directly to the carriers (direct covalent immobilization) or bind indirectly to the carriers at the flexible “arm” (spacer) (indirect covalent immobilization) [40]. In both cases, the first step of immobilization involves chemical modification of the carrier groups to provide covalent binding of the enzymes (direct covalent immobilization) or the spacer “arm” on the carrier, followed by activation of the spacer “arm” for covalent binding of the enzyme (indirect covalent immobilization). Chemical modification of the carrier groups can be carried out with tresyl chloride, sulfonyl chloride, bromocyanine, epichlorohydrin, glutaraldehyde, N-hydroxy-succinimidyl, sodium periodate, hydrazine, ß-mercaptoethanol, and dithiothreitol, while glutaraldehyde, hexamethylenediamine, and polyethylenimine are used as spacer arms [47]. In addition, crosslinking is another type of covalent immobilization in which enzymes are crosslinked in solution to form aggregates, or crosslinking occurs after adsorption.

The entrapment method is based on the following: (i) entrapment of an enzyme in a polymeric network that allows the substrate and products to pass through but retains the enzyme, (ii) entrapment of enzymes in organelle-like structures, and (iii) anchoring of enzymes in a phospholipid layer [35,40,47,48,49,50,51,52,53,54,55,56,57,58].

Considering the advantages and disadvantages of the above-mentioned types of enzyme immobilization in terms of their cost and simplicity, it can be said that adsorption is the cheapest and simplest method of immobilization, while immobilization by covalent binding as well as immobilization by enzyme entrapment is much more complicated and therefore more expensive. On the other hand, immobilization by adsorption is usually the worst in terms of stability and reusability of immobilized enzymes, because immobilized enzymes may leak from the carrier (“carrier leakage”), which is rare in immobilization by enzyme entrapment and extremely rare in immobilization by covalent binding. Therefore, the reusability of more tightly immobilized enzymes is higher. In addition, covalent binding usually leads to a high proportion of the bound enzyme on the carrier and enables the reusability of the immobilized enzyme. However, direct covalent binding usually leads to changes in enzyme conformation, often caused by the significantly lower activity of the immobilized enzyme compared to the free enzyme, which can be partially corrected by indirect covalent immobilization, where flexible chemical structures reduce steric interference between enzyme molecules and allow for greater mobility [35,40,48,49,51,55,58]. All of the above methods can be generally applied to the immobilization of lipases.

Physical compressive strength, ease of derivatization, chemical inertness to the enzyme and the environment in the reaction mixture, biocompatibility, biodegradability, nontoxicity, availability, and very low cost are the preferred properties of carriers for enzyme immobilization [2,51,54]. For the immobilization of enzymes, commercially available organic or inorganic carriers of natural or synthetic nature are usually used, which in most cases meet the desired properties of the carrier. However, most commercially available carriers are expensive, which significantly increases the cost of preparing immobilized enzymes. Therefore, there is a need to find cheaper carriers for immobilization, such as widely available waste/by-products from the agri-food industry. Moreover, in lieu of the substantial cost of waste disposal, the agri-food industry might even realize some profit and make a substantial long-term gain by investing in the production of immobilization carriers.

Among the numerous wastes generated in the agri-food industry, eggshells, coffee grounds, and brown onion skins are particularly “suitable” candidates (raw materials) for the preparation of carriers for the immobilization of enzymes. The latter is supported by the available information on their structure, chemical composition, and current knowledge on their potential use. Treatment of these wastes could result in collagen-based carriers (eggshell membrane) and cellulose- and/or hemicellulose-based carriers (spent coffee grounds and brown onion skins). Unlike other wastes from the agri-food industry (residues after processing of cereals, corn, oilseeds, sugar beet bagasse [59,60,61], rice [62], coconut [63], residues from the wood processing industry, etc.), which are widely used in biorefineries as feedstock for the production of high-value products such as chemicals, materials, biofuels, and bioenergy [64], eggshells, spent coffee grounds, and brown onion skins have not yet found a suitable place in sustainable production and are mainly landfilled and thus represent an additional burden on the environment.

### 2.1. Carriers Based on Eggshell Membranes

The average annual production of eggs is 65 million tones, of which about half is used in industrial production, resulting in 6.5 million tones of industrial waste to be disposed of [65]. Moreover, according to an estimate of the increase of global egg production to 90 million tones per year by 2030 [66], the amount of industrial waste that needs to be properly disposed of is also expected to increase. In the European Union, about 11 million tones of eggs are produced annually, of which about 37 thousand tones are produced in the Republic of Croatia (RH).

Eggshells represent about 11% of the total egg mass and contain on average 94% calcium carbonate, 1% magnesium carbonate, 1% calcium phosphate, and 4% organic matter [66,67].

**Figure 3:**
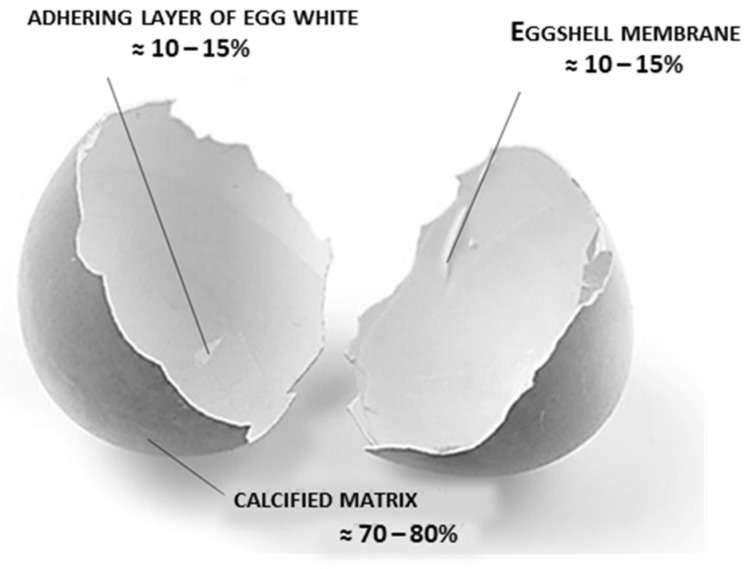
Structure and size ratios of the individual structural components of the eggshell (modified according to Ref. [77], with permission from publisher Josip Juraj Strossmayer of Osijek, Faculty of Food Technology Osijek, 2021).

Structurally (Figure 3), eggshell wastes contain the following two main parts: the calcified matrix (shell) and the organic envelope, called eggshell membrane in the available literature. The calcified matrix is dominated by calcium carbonate in the form of calcite crystals, whereas the eggshell membrane is dominated by collagen in the form of cross-linked protein fibres [68,69]. The eggshell membrane consists of two tightly bound membranes (one inner and one outer), which, because of their chemical composition, resemble the basal lamina or extracellular cement of proteins, lipids, and carbohydrates, to which cells are bound during tissue coupling. In addition to the calcified matrix and membrane, the eggshells also contain some of the proteins remaining on the membrane and some number of microorganisms. According to the available literature data, the removal of protein from eggshells is achieved by cooking and/or washing with distilled water [65,70,71,72,73,74] or boiling in a 0.1% sodium dodecyl sulphate solution [71]. Cleaning of eggshells and removal of microorganisms is achieved by using dilute acid solutions such as acetic and hydrochloric acids. These procedures are most commonly used in the preparation of eggshells as a carrier for the immobilization of enzymes [65,70,71,72,73,75,76]. However, the available literature does not provide data on the effect of different eggshell pretreatment methods (i.e., cooking in 0.1% sodium dodecyl sulphate solution or washing in acidic solutions) on enzyme immobilization efficiency.

**Figure 4:**
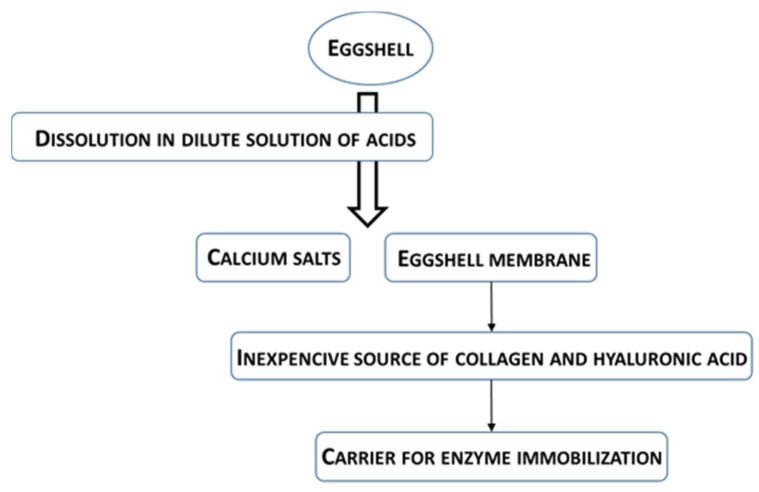
Schematic representation of eggshell transformation into calcium salts and membrane.

Research by Ribeiro et al. [78], Salleh et al. [75], Chattopadhyay and Sen [79], Norouizan et al. [76], Vemuri et al. [71], Venkaiah and Kumar [80], and Makkar and Sharma [70] indicates the potential of eggshells as carriers for enzyme immobilization after some degree of pretreatment. Immobilization of enzymes (urease, lipase, and tyrosinase) on eggshells was performed by adsorption [75], adsorption by sequential entrapment of the enzyme in a glutaraldehyde matrix [70,71], and adsorption on a polyethyleneimine-coated shell followed by entrapment of the enzyme with glutaraldehyde [76]. However, it is unlikely that they will find their way into commercial production as carriers in their intact form. The main reason for this is that the calcified matrix of eggshells is a very cheap source of natural calcium in the form of calcium carbonate, from which inorganic and organic calcium salts can be prepared by relatively simple processes. Organic and inorganic calcium salts are used in the food, pharmaceutical, and chemical industries (as additives and dietary supplements), as well as for the production of calcium-based fertilizers for agricultural purposes [66,67,81,82]. Among the numerous methods of obtaining calcium salts from eggshells, the best known are those based on dissolving calcium carbonate from the calcified matrix in dilute solutions of acids such as hydrochloric, acetic, and o-phosphoric acids. Dissolution with acids also produces the eggshell membrane as a byproduct (Figure 4). Eggshell membrane is not only a cost-effective source of collagen and hyaluronic acid for the pharmaceutical and cosmetic industries [66,83], but unlike the calcified matrix, it has great potential to be used as a carrier for enzyme immobilization [65,72,83,84,85,86]. This is supported by many studies focused on the development of biosensors based on the immobilization of enzymes on an eggshell membrane as solid carrier [73,83,87,88,89,90,91,92,93,94,95,96].

Comparing the production of calcium salts by treating eggshells with dilute acids with the production of eggshell membranes for the development of biosensors, it can be seen that ground eggshells are used for the production of salts while intact eggshells or eggshell halves are used for the production of eggshell membranes for biosensors. Moreover, in most cases of eggshell membrane preparation for biosensor development, partial decalcification of eggshells by acids is performed to facilitate the separation of large portions of eggshell membrane from the remaining eggshells. Accordingly, it seems very likely that these two types of production processes could be combined to simultaneously produce calcium salts and larger pieces of the eggshell membrane by using half and/or larger pieces of eggshells instead of ground ones. This would greatly facilitate the eggshell membrane separation and handling process. In addition, the eggshell membrane produced could be uniformly ground to the particle size required for the preparation of carriers for lipase immobilization. One of the most important facts related to the separation of eggshell membranes from eggshells by acids is that exposure of eggshell membranes to acids leads to changes in their chemical composition (compared to the untreated membrane) [97,98]. However, there are no data in the literature to date discussing the effects of various acid-induced changes in eggshell membrane composition on enzyme immobilization efficiency. Considering that changes in the chemical composition of the carrier can alter the immobilization efficiency, such studies would allow the selection of the most appropriate eggshell membrane preparation process to obtain the carrier with the desired properties.

### 2.2. Carriers Based on Spent Coffee Grounds and Brown Onion Skins

The European Union is one of the largest consumers of coffee in the world [96]. Annual imports of coffee beans (raw and roasted) into the EU amount to almost 5 million tones, of which the Republic of Croatia accounts for about 23 thousand tones. If we take into account the fact that about half of a coffee bean is consumed in the production of liquid beverages that produce coffee grounds [99,100], it becomes clear that coffee grounds are a widely available raw material. This is supported by the fact that approximately 6 million tones of coffee grounds are produced annually worldwide [100]. The average annual production of onions (*Allium cepa* L.) is about 66 million tones [101], with about 10.5 million tones produced in the European Union and about 19 thousand tones in the Republic of Croatia [102]. Onion processing generates significant amounts of waste, estimated at 500,000 tones annually in the EU [101].

**Figure 5:**
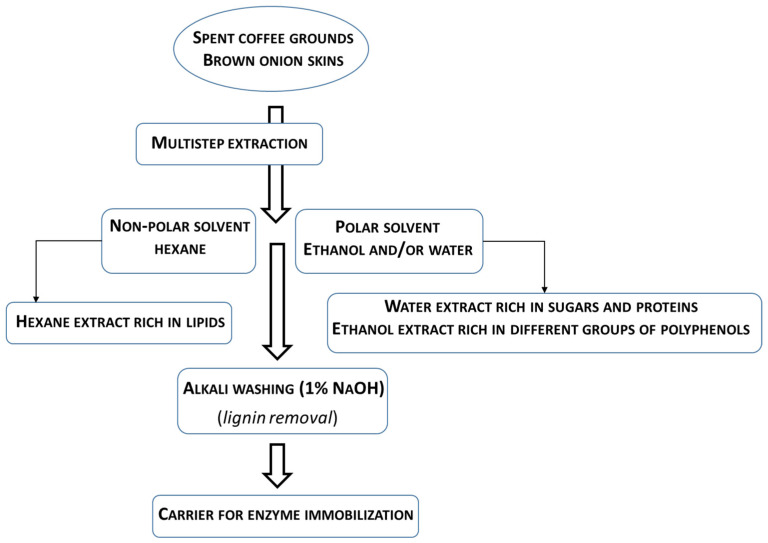
Schematic representation of the transformation of spent coffee grounds and brown onion skins into high-value biological compounds and lignocellulosic material.

Both the spent coffee grounds and the brown onion skins have complex chemical compositions [99,100,101,103,104,105,106,107,108]. According to literature reports, spent coffee grounds contain about 60% water-insoluble lignocellulosic material, i.e., about 50% insoluble fibres in dry matter, with cellulose and hemicellulose dominating the fibres [99,100,104,107,109,110,111], while brown onion skins contain about 60% insoluble fibre in dry matter, with the major polysaccharide of insoluble fibre being cellulose [101,105,106,108,112,113]. In addition to the predominant lignocellulosic polymers—cellulose and hemicellulose, which are mainly responsible for the suitability of these waste materials as enzyme carriers, they also contain certain amounts of proteins, polyphenols, and lipids [99,100,101,103,104,105,106,107,108,109,110,111,112,113,114] that can interfere with the process of enzyme immobilization and must be removed by extraction. For example, coffee grounds obtained from the industrial preparation of instant coffee contain between 13.6 and 17.5% proteins, 10 and 15% lipids, and about 4% polyphenols and 2.5% condensed tannins in dry matter [99,100,104,107]; brown onion skins contain from 2 to 5% protein, about 1% lipid, and about 5.3% polyphenols [101,103,105,106,114]. Since the above components differ in polarity and thus extractability in different solvents [101,104,105,106,107,115], a multistep extraction system is required as a pretreatment procedure for the preparation of suitable chemically inert carriers for enzyme immobilization based on cellulose or mixtures of cellulose and hemicellulose, including a nonpolar solvent (hexane), followed by a polar solvent (ethanol and/or water), and finally an alkaline wash (1% NaOH) (Figure 5).

In addition to the chemically inert lignocellulosic material, extracts enriched with specific groups of high-value bioactive components are expected, including certain oils and terpenes, polyphenols and flavonoids, and proteins. For example, the lipids from spent coffee grounds and brown onion skins should be concentrated in the hexane extract, the simple sugars and proteins should be concentrated in the water extract and the 1% sodium-based extract, and the various groups of polyphenols should be concentrated in the ethanol and water extracts and the 1% sodium-based extract. In any case, the process of multistep extraction must be optimized in terms of starting material (spent coffee grounds, brown onion skins), extraction time, and polar solvent (two-solvent system ethanol and water or single solvent ethanol or water). These products could be further used to produce other high-value products based on an analysis of their chemical composition.

As mentioned above, the suitability of spent coffee grounds and brown onion skins as carriers for the immobilization of enzymes is mainly due to the presence of lignocellulosic polymers. Cellulose is a long, unbranched polymer of glucose units linked by *β*-1,4-glycosidic bonds. It is the main component of the plant cell wall and the most abundant polymer in nature. Cellulose is insoluble in water, chemically inert under mild reaction conditions of enzymatic reactions, non-toxic, and biodegradable. Cellulose fibers are resistant to mechanical shear forces. In addition, due to the hydroxyl groups present on the surface of cellulose fibers, cellulose is a suitable carrier for the immobilization of enzymes by adsorption, but can also serve as an inexpensive carrier that can be used for covalent binding of the enzyme after certain chemical modifications. Furthermore, to commercially available forms of pure cellulose and its modified derivatives, water-insoluble lignocellulosic material from agri-food industry waste can also be used as an even more cost-effective carrier for lipase immobilization. This can be demonstrated by research on immobilization of lipases on olive pomace [116,117,118], rice husks [53,57], corn stalks [119], coconut shells or fibers [119,120], palm stalks [119], and loofah sponges [121]. Immobilization of lipase was achieved by adsorption to pretreated and processed waste or by covalent binding to the chemically modified carrier (e.g., chemically modified pretreated waste material). The two most commonly used lipases in immobilized form are from *Thermomyces lanuginosus* (TLL) and *Candida antarctica* lipase B (CALB). Covalent immobilization methods were used to immobilize lipases on olive pomace [113,114,115] and rice husks [53] using glutaraldehyde to activate carriers from the aforementioned wastes. Cespugli et al. [57] prepared rice husks for covalent binding in a different way, first oxidizing them and functionalizing them by introducing a di-amino spacer. CALB lipase immobilized in this way showed better results in terms of operational stability compared to the same lipase immobilized on a commercial carrier, methacrylic resins. *T*. *lanuginosus* lipase immobilized on olive pomace [116,117,118] showed operational stability in a functional test reaction (synthesis of biodiesel from pomace oil with methanol) in up to 10 consecutive batches. Corici et al. [53] and Brigida et al. [119] immobilized lipase by adsorption on rice husks and coconut fibers, respectively, while Ittrat et al. [120] used corn stalks, palm stalks, coconut shells, corn cobs, rice husks, *Wodyetia bifurcata* AK Irvine leaves, and *Salacca wallichiana* stems. The worst results were reported by Corici et al. [53], probably due to the low activity of immobilized lipase and low operational stability. On the other hand, the results of Brigida et al. [119] showed that the immobilized lipase (CALB) exhibited increased temperature stability up to 50–60 °C and retained its activity up to 50% after three cycles in the functional test reaction with methyl butyrate hydrolysis. Similarly, in the second reaction with butyl butyrate synthesis, the immobilized CALB retained its activity up to 80% over six cycles. The results reported by Ittrat et al. [120] showed that the carrier based on *Salacca wallichiana* stem proved to be the best among all other carriers investigated. Compared with the immobilized free lipase, the storage stability at room temperature (25–32 °C) and the stability to organic solvents increased, but a significant loss of activity was observed after two consecutive cycles of use, which was due to the leakage of the enzyme from the carrier.

The above examples of immobilization of lipase on various carriers derived from waste from the agri-food industry refer to carriers based on lignocellulosic material. While cellulose is a linear polymer composed of glucose molecules, hemicellulose is a branched polymer composed of various monosaccharides (pentoses and hexoses), depending on the plant species. Lignin, on the other hand, is a polymer composed of various propyl phenol units, namely, coniferyl alcohol, sinapyl alcohol, and coumaryl alcohol, as well as other modified phenolic acids. It is important to emphasize that both types of polymeric material, hemicellulose and lignin, surround the cellulose fibers in plant cell walls [99,100,104,107]. Accordingly, it is expected that during immobilization of lipase on the lignocellulosic material obtained by pretreatment of spent coffee grounds or brown onion skins (depending on the type of pretreatment), multiple interactions will occur between the side branches of the enzyme and the functional groups of the lignocellulosic material. Therefore, in the process of waste preparation as a carrier for lipase immobilization, it is necessary to remove lignin. To obtain lignocellulosic carriers from spent coffee grounds and brown onion skins, sequential solid-liquid extraction with hot solvents must be performed. The sequential extraction from non-polar to polar solvents involves the non-polar solvent n-hexane, followed by two polar solvents (water and ethanol), after which the preparation of the carrier must be completed by alkaline washing (1% aqueous sodium hydroxide solution) as the final step for lignin removal [107,115].

There are few publications on the possibility of using spent coffee grounds and brown onion skins as potential carriers for enzyme immobilization. In the case of brown onions, there are only three papers that can be put in context since the immobilization occurred on the inner membrane of the red onion. Kumar and Pundir [122] covalently immobilized lipase from porcine pancreas on the onion membrane by previously activating it with glutaraldehyde. The functionality test reaction was milk hydrolysis with the aim of producing skim milk. The activity of immobilized lipase was 63.6% of the initial value. The immobilized lipase was used up to 100 times over two months without significant loss of activity when stored at +4 °C. Authors Kumar and D’Souza [123] and Wang et al. [124] also chose the brown onion membrane as an immobilization carrier for the preparation of biosensors for the detection of glucose in model solutions and real systems, fruit juices, and wines. The immobilized enzyme was glucose oxidase, and the resulting biosensor proved to be very effective and stable. Kumar and D’Souza [123] used the biosensor up to 127 times, and the immobilized glucose oxidase retained up to 90% of its original activity. However, this raises the question of the cost-effectiveness of the manufacturing process of the biosensor on a support that is the inner membrane of the brown onion, since the brown onion membrane is an edible part of the onion and not a waste product in the processing of the brown onion. The same can be observed in the case of spent coffee grounds as a potential carrier for enzyme immobilization, for which there are only four literature data points [125,126,127,128]. Chen et al. [125] immobilized *β*−glucosidase by covalent binding to spent coffee grounds activated with glutaraldehyde and used it to convert isoflavone glycosides to their aglycones from black soy milk. The immobilized *β*−glucosidase was stable for 20 days without losing activity in 30 batches. In their work, Buntić et al. [126,127,128] performed immobilization of cellulase by adsorption and covalent binding. The results indicate an improvement in storage stability and activity of immobilized cellulase compared to its free form. The functionality test reactions with immobilized cellulases were not performed in their studies.

In all four cases, enzyme immobilization was performed on carriers with questionable properties, as they were prepared without complete removal of all possible components that could interfere with the immobilization process. One of the most important properties of the carriers is their chemical inertness towards the enzyme and the reaction mixture. Therefore, it is necessary to apply various treatment procedures to obtain carriers with these properties. The systematic analysis of the composition of the starting wastes, of the intermediates in the preparation of the carrier and finally of the carriers is necessary to complete the lack of knowledge about the possible application of these three discussed wastes.

## 3. Conclusions and Future Directions

Biocatalytic processes generally leave a barely noticeable environmental footprint compared to chemically catalyzed processes. They are more environmentally friendly and more in line with sustainable development principles. Therefore, future research on the development of heterogeneous biocatalysts based on agricultural and food wastes will certainly have a significant impact and contribution in the field of lipase immobilization and waste utilization. It will also help to fill the knowledge gap mentioned above regarding the reuse of the wastes discussed in this review, i.e., eggshells, coffee grounds, and brown onion skins as carriers, and clarify the lipase immobilization processes. Based on all the above, it is obvious that a systematic analysis of the usability of eggshells, spent coffee grounds, and brown onion skins as carriers for enzyme immobilization is needed and should include the following: (i) different types of carrier preparation, (ii) different techniques for lipase immobilization, (iii) evaluation of immobilization efficiency in terms of the amount and activity of the immobilized enzyme, operational stability, and rate and production yield of the desired end products, and (iv) reusability of the immobilized lipases in the production process.

Considering that agri-food wastes are widely available and inexpensive, their use is expected to have a positive long-term impact on the development of society and the economy by reducing environmental pollution, lowering the price of immobilized lipases, and ultimately achieving a potentially lower price for the final products obtained in a healthy and safe manner through biocatalytic processes. In addition, agri-food industry waste is recycled, which is one of the cornerstones of the circular economy. Lipases immobilized on agri-food industry wastes represent a technological and economic improvement of biocatalytic production, indicating the possibility of achieving sustainable production based on the circular economy and approaching the “zero waste” model.

## Data Availability

The datasets generated for this study are available on request to the corresponding author.
